# Biodistribution and radiation dosimetry of the PDGFRβ targeting PET tracer [^68^Ga]Ga-ATH001

**DOI:** 10.1007/s00259-026-08167-9

**Published:** 2026-09-08

**Authors:** Mark Lubberink, Talakad G. Lohith, Zoltan Derdak, Paul Wilkens, Emilia Palmér, Alexandra Ramolli, Mélodie Ferrat, Thuy A. Tran, Bogdan Mitran, Ayman Abouyzayed, Lars Johansson, Haakan Wennbo, Fredrik Rorsman, Johan Vessby, Olof Eriksson

**Affiliations:** 1 Molecular Imaging and Medical Physics, Department of Surgical Sciences, Uppsala University, Uppsala, Sweden; 2 Takeda Development Center Americas, Inc, Cambridge, MA USA; 3 Department of Molecular Medicine and Surgery, Karolinska Institutet, Stockholm, Sweden; 4 Department of Nuclear Medicine and Medical Physics, Karolinska University Hospital, Stockholm, Sweden; 5 Department of Oncology and Pathology, Karolinska Institutet, Stockholm, Sweden; 6 Theranostics Trial Center, Karolinska Institutet, Stockholm, Sweden; 7 Antaros Tracer AB, Uppsala, Sweden; 8 Antaros Medical AB, Gothenburg, Sweden; 9 Department of Gastroenterology and Hepatology, Uppsala University Hospital, Uppsala, Sweden; 10 Department of Medical Sciences, Gastroenterology Research Group, Uppsala University, Uppsala, Sweden; 11 Department of Medicinal Chemistry, Science for Life Laboratory, Uppsala University, Dag Hammarskjölds Väg 14 C, 3Tr SE-751 83, Uppsala, Sweden

**Keywords:** PET, Fibrosis, Fibrogenesis, Dosimetry, PDGFRβ

## Abstract

**Purpose:**

[^68^Ga]Ga-ATH001 is an Affibody molecule targeting PDGFRβ in development as a Positron Emission Tomography (PET) tracer for non-invasive imaging of active fibrosis. Here we present first-in-human [^68^Ga]Ga-ATH001 biodistribution and radiation dosimetry data.

**Methods:**

Radiation dosimetry of [^68^Ga]Ga-ATH001 was evaluated in healthy participants (n = 3) and participants with metabolic dysfunction-associated steatohepatitis (MASH) (n = 3). Each participant was intravenously administered ~ 2.5 MBq/kg [^68^Ga]Ga-ATH001 and imaged by whole-body PET for up to 3 h post-injection. Absorbed dose coefficients and effective doses according to ICRP 103 were calculated using IDAC DOSE 2.1.

**Results:**

The effective dose of [^68^Ga]Ga-ATH001 was 0.028 ± 0.002 mSv/MBq with kidneys as the dose-limiting organ. Administration of a target dose of 2 MBq/kg would result in a sex-averaged effective dose of 4.1 mSv. Subjects tolerated the tracer administration well.

**Conclusion:**

[^68^Ga]Ga-ATH001 demonstrates a dosimetry and safety profile that supports further clinical development as a repeatable, non-invasive PET marker of active fibrosis in tissue.

**Supplementary Information:**

The online version contains supplementary material available at https://doi.org/10.1007/s00259-026-08167-9.

## Introduction

Fibrosis and tissue remodeling are key hallmarks of chronic inflammatory diseases and fibrotic disorders. These processes involve excessive deposition of extracellular matrix (ECM) components, such as collagen, leading to scarring, tissue hardening, and disrupted architecture. Fibrosis arises from persistent tissue injury or inflammation, where normal wound healing becomes dysregulated. Tissue fibrosis is a central driver in diseases of diverse etiologies, including metabolic dysfunction-associated steatohepatitis (MASH).

Platelet Derived Growth Factor Receptor Beta (PDGFRβ) is a broadly accepted marker of pericytes and is associated with a wide range of fibrotic pathologies and tissue remodeling [1]. PDGFRβ is strongly upregulated on activated hepatic stellate cells, the main source of extracellular matrix deposition in liver fibrosis. Targeting PDGFRβ is therefore a potential strategy for non-invasive Positron Emission Tomography (PET) imaging of hepatic disease severity and active fibrogenesis [2].

[^68^Ga]Ga-DOTA-Cys-ATH001 ([^68^Ga]Ga-ATH001, also referred to in literature as Z09591) is an Affibody molecule in development as a PET tracer for imaging of fibrogenesis by quantification of PDGFRβ in tissue [3], e.g. in liver [4, 5], lung [6], heart ([7, 8], NCT06956560) and tumor stroma [9].

Here, we present first-in-human biodistribution and radiation dosimetry data of [^68^Ga]Ga-ATH001 in healthy participants and participants with MASH in a clinical PET study (NCT06562361).

## Methods

### Clinical study design and participant demographics

Radiation dosimetry of [^68^Ga]Ga-ATH001 was evaluated in two sub-cohorts of an ongoing first-in-human clinical study NCT06562361 [10] : Healthy participants (cohort 1a, n = 3, 1 male, all 22 years old, 64–73 kg) and participants with MASH (cohort 2a, n = 3, 3 males, 54–65 years, 75–108 kg). The study was approved by the Swedish Medical Products Agency and the Swedish Ethical Review Authority (approval number 2023–506555-15–00) and was conducted in consistency with Good Clinical Practice and the Declaration of Helsinki. All participants underwent safety monitoring before, during and after dosing, including but not limited to laboratory safety assessments, vital signs and electrocardiography.

### PET scanning protocol

A dynamic PET/MRI (Signa, GE Healthcare) scan over the abdomen (0–35 min p.i.) was started simultaneously with intravenous bolus administration of a target dose of 2.5 MBq/kg [^68^Ga]Ga-ATH001 (178 ± 25 MBq, corresponding to 51 ± 10 µg peptide), followed by whole-body scans at 1, 2 and 3 h (3, 4 and 5 min/bed position respectively) post-injection. The molar activity at time of injection was 25.1 ± 3.6 MBq/nmol. The PET scanner was cross calibrated with the dose calibrator at regular intervals to ensure accurate quantification of activity concentrations. All appropriate corrections were applied, and images were reconstructed using time-of-flight ordered subset expectation maximisation (4 iterations, 28 subsets) with point spread function recovery and a 4 mm plain gaussian post-filter. Attenuation correction was based on the 2-point Dixon method. A total of 25 organs and tissues were segmented for dosimetry assessment, namely liver, kidneys, heart wall, lungs, spleen, red bone marrow, descending aorta, salivary glands, thyroid, prostate gland, cortical bone, trabecular bone, pancreas, gallbladder, urinary bladder contents, breast including skin, muscle, stomach content, brain, testes, ovaries, uterus, small intestines (SI) contents, upper large intestines (ULI) contents, lower large intestines (LLI) contents. See Supplementary Table S1 for details on the methods of segmentation for each tissue.

### Time-integrated activity coefficients (TIACs)

All time-activity curves (TACs) were analyzed without decay correction. The area under the curve (AUC) of the dynamic imaging portion of each TAC was calculated by rectangular integration, i.e. multiplication of the value in each frame of the dynamic scan with the corresponding frame duration. A single exponential fit to the last data point in the dynamic scan and the three whole-body scans were used to estimate the remainder of the AUC by analytical integration to infinity. For source organs for which only data from the three whole-body scans was available, a single exponential fit to the three whole-body scan data points was used to estimate the entire AUC. The time-integrated activity was calculated as the AUC under each TAC. The AUC was multiplied by its respective organ weight by proportional scaling of the adult male or female reference organ weights in ICRP110 to each participant’s body weight relative to the ICRP110 total body weights. This assumes identical standardized uptake values (SUVs) between individual subject and the reference phantom. Time-integrated activity coefficients (TIACs) were calculated by division with the amount of injected activity. Remainder of the body TIAC was calculated as the theoretical maximum residence time minus all source organ residence times. Muscle was included in remainder of the body. No urinary bladder voiding was assumed.

### Absorbed dose calculations

Absorbed dose coefficients were calculated using IDAC DOSE 2.1 (https://www.idac-dose.org/idac-dose) [11]. Sex-averaged effective dose was calculated according to ICRP103 [12].

## Results

### Tracer biodistribution and clearance

**Fig. 1:**
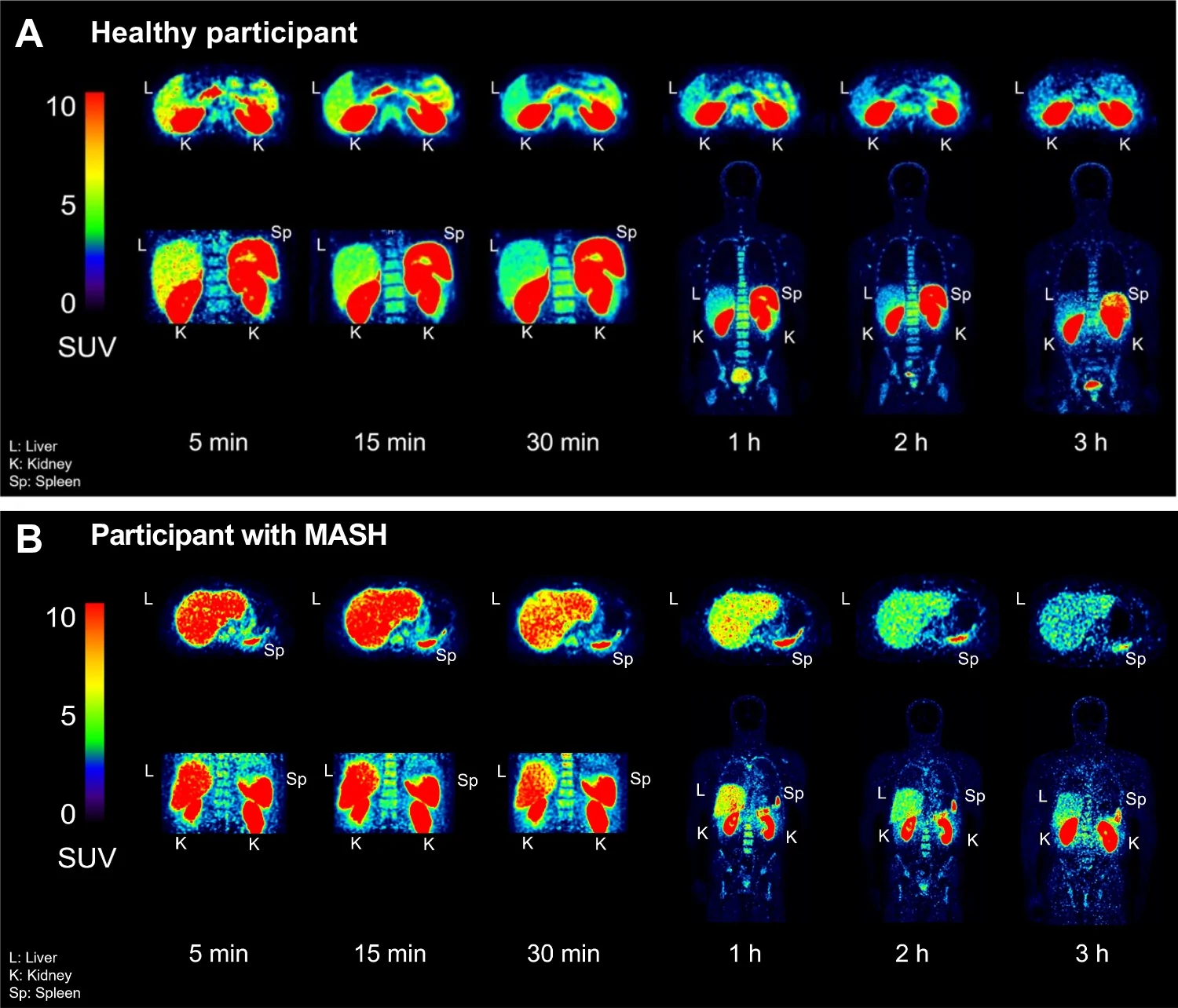
[^68^Ga]Ga-ATH001 PET images of a typical healthy participant (**A**) and a participant with MASH (**B**). Transaxial projections from the initial dynamic abdominal scan, and coronal projections of the subsequent WB scans are shown. Images are decay corrected to the time of injection and normalized to SUV 10. L = liver, K = kidney, SP = spleen

[^68^Ga]Ga-ATH001 demonstrated rapid biodistribution, followed by clearance from most tissues in both healthy participants (Fig. 1A) and participants with MASH (Fig. 1B). Two hours after administration, only uptake in kidneys and bladder (main excretion route) as well as PDGFRβ-avid spleen remained, while e.g. liver approached background in healthy participants. Liver uptake was higher in participants with MASH (SUV 4.0 ± 0.6) compared to healthy participants (SUV 2.8 ± 0.1) two hours after tracer administration (p < 0.05, unpaired t-test).

### Dosimetry calculations

**Fig. 2:**
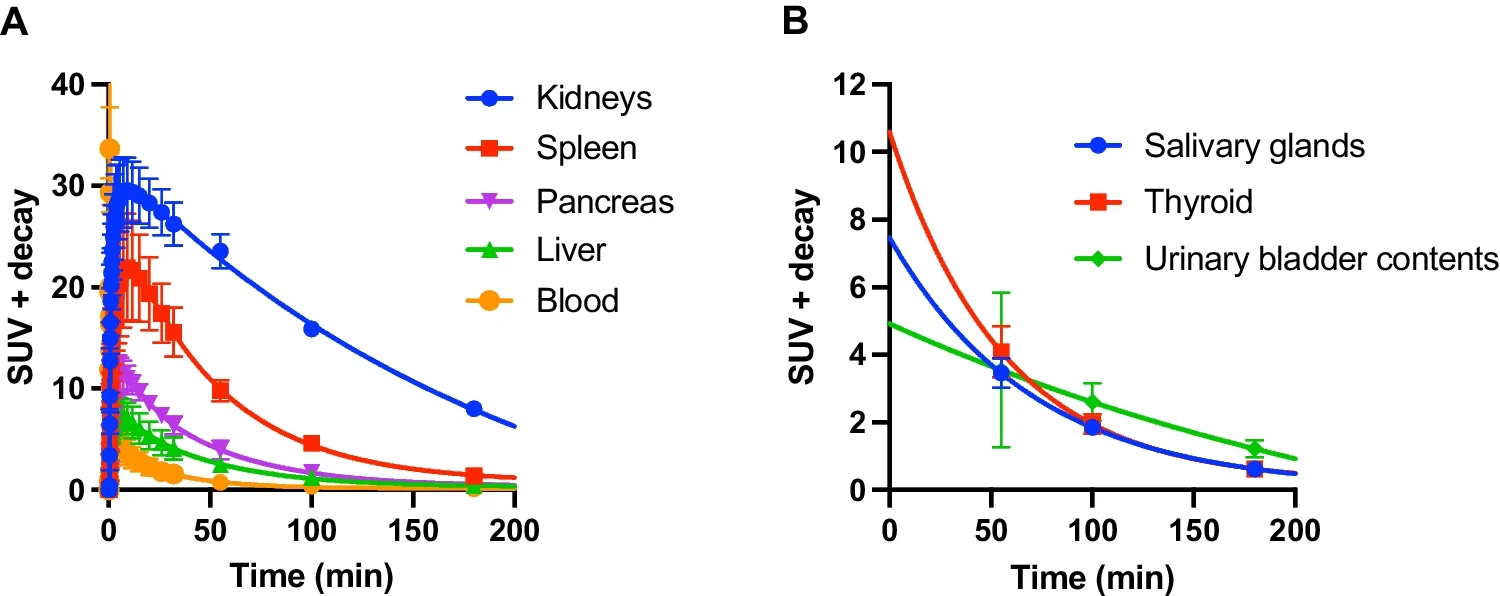
SUV (including radioactive decay) versus time in organs with highest uptake in the FOV of the initial dynamic scan over the abdomen (**A**) and tissues outside of the FOV of the dynamic scan (**B**). Error bars indicate standard deviations. The solid lines after 32 min are single-exponential fits to the data

Tissue uptake was quantified as SUV over time (without decay correction for further processing). Organs with highest initial uptake included e.g. kidneys, spleen, pancreas, liver and blood (Fig. 2A-B). Effective half-lives based on the whole-body scan data and the percentages of the TIACs that are due to extrapolation beyond the last measured data point are presented in Supplementary Table S2. TIACs for all source organs are shown in Supplementary Table S3.

The sex-averaged effective dose of [^68^Ga]Ga-ATH001 was 0.028 ± 0.002 mSv/MBq (Table 1). The dose-limiting organ was the kidneys (0.34 ± 0.03 mGy/MBq in males and 0.46 ± 0.02 mGy/MBq in females). A target activity of 2 MBq/kg (147.4 MBq in males with a weight of 73.7 kg per ICRP103), would result in a sex-averaged effective dose of 4.1 mSv and an absorbed dose to the kidneys of 50.1 mGy.

**Table 1:** Absorbed and effective dose coefficients for [^68^Ga]Ga-ATH001

Organ	Female (N = 2) (mean ± SD, mGy/MBq)	Male (N = 4) (mean ± SD, mGy/MBq)
Adrenals	0.055 ± 0.002	0.062 ± 0.005
Brain	0.002 ± 0.000	0.002 ± 0.000
Breast	0.007 ± 0.000	0.008 ± 0.000
Colon wall	0.014 ± 0.001	0.013 ± 0.001
Endosteum (bone surfaces)	0.015 ± 0.001	0.013 ± 0.001
ET region	0.006 ± 0.000	0.005 ± 0.000
Eye lenses	0.005 ± 0.000	0.004 ± 0.001
Gallbladder wall	0.053 ± 0.003	0.038 ± 0.005
Heart wall	0.030 ± 0.003	0.027 ± 0.005
Kidneys	0.463 ± 0.021	0.339 ± 0.033
Liver	0.055 ± 0.001	0.051 ± 0.012
Lungs	0.017 ± 0.001	0.014 ± 0.002
Lymphatic nodes	0.018 ± 0.000	0.013 ± 0.000
Muscle	0.011 ± 0.000	0.009 ± 0.001
Oesophagus	0.013 ± 0.000	0.012 ± 0.000
Oral mucosa	0.009 ± 0.000	0.007 ± 0.001
Ovaries	0.022 ± 0.000	
Pancreas	0.092 ± 0.000	0.074 ± 0.011
Prostate		0.040 ± 0.006
Red marrow	0.028 ± 0.002	0.022 ± 0.001
Salivary glands	0.061 ± 0.003	0.046 ± 0.005
Skin	0.007 ± 0.000	0.006 ± 0.001
SI wall	0.026 ± 0.000	0.019 ± 0.004
Spleen	0.161 ± 0.016	0.119 ± 0.015
Stomach wall	0.027 ± 0.001	0.023 ± 0.005
Testes		0.015 ± 0.003
Thymus	0.012 ± 0.000	0.010 ± 0.001
Thyroid	0.059 ± 0.009	0.062 ± 0.005
Urinary bladder wall	0.034 ± 0.008	0.026 ± 0.004
Uterus	0.064 ± 0.004	
Effective dose coefficient (mSv/MBq), ICRP 103	0.028 ± 0.002	

There were no statistically significant differences in absorbed dose in individual tissues nor the effective dose between healthy participants and participants with MASH.

### Safety profile

Subjects were monitored for up to 24 h post-dosing. All participants tolerated the tracer well with no recorded adverse effects following tracer administration.

## Discussion

There is an urgent and unmet need for non-invasive assessment of active tissue fibrosis, for improved diagnosis and patient stratification, as well as longitudinal monitoring of novel anti-fibrotic interventions. PDGFRβ activity is closely linked to critical physiological and pathophysiological processes including angiogenesis, fibrogenesis and tissue remodeling.

[^68^Ga]Ga-ATH001 binds to human PDGFRβ with picomolar affinity [3] and is in clinical development as a general PET marker of active fibrogenesis in several indications.

Here, we present first-in-human [^68^Ga]Ga-ATH001 whole-body biodistribution data, demonstrating low background and rapid clearance from most tissues, including organs often afflicted by fibrosis, such as liver, heart and lung. The sole route of excretion was through kidney and bladder, just like for the elimination of similar gallium-68 radiolabeled, DOTA-conjugated Affibody molecules evaluated in human [13]. The intense uptake in the kidney cortex is due to reabsorption of larger peptide-based radiotracers by the renal tubules, followed by intracellular trapping of ^68^Ga. This was reflected by the kidneys being the dose-limiting organ for [^68^Ga]Ga-ATH001.

Uptake in liver was higher in participants with MASH compared to healthy participant. This is likely due to the upregulation of PDGFRβ on activated hepatic stellate cells due to ongoing liver fibrogenesis in the participants with MASH. The uptake of [^68^Ga]Ga-ATH001 in liver will be further explored in additional participants with different stages of MASH in the ongoing clinical study NCT06562361.

The main sources of uncertainty in the reported TIACs are the extrapolation of the measured data and the scaling of activity concentration in representative parts of organs to whole organ activity. The scan protocol, consisting of a 35 min dynamic scan followed by whole-body imaging up to 3 h p.i., minimized the need for extrapolation of the data with the extrapolated fraction beyond the last measurement point remaining below 20% in most organs included in the field of view of the dynamic scan (Supplementary Table S2). The uncertainty is higher in organs not included in the dynamic portion of the scan, but none of these makes a major contribution to the effective dose. The urinary bladder contents TIAC was based solely on the PET data, which likely results in an overestimation of the TIAC and, consequently, the absorbed dose. The effective half-life was lower than the physical half-life of ^68^Ga in all organs except brain, breast, kidneys, testes, uterus and urinary bladder contents, reflecting continuing tracer accumulation and slow renal clearance.

The effective dose was 0.028 ± 0.002 mSv/MBq, which is comparable to other ^68^Ga-labeled Affibody molecules [14] or slightly higher than for established ^68^Ga peptide-based tracers such as [^68^Ga]Ga-DOTATOC [15] or the most common PET radiopharmaceutical ^18^F-fluorodeoxyglucose ([^18^F]FDG).

Administration of a target activity of 2 MBq/kg (approximatively 150 MBq in males) would result in a sex-averaged effective dose of 4.1 mSv and an absorbed dose to the kidneys of 50.1 mGy in males, with slightly higher doses (55.2 mGy) in females. Thus, the dosimetry profile of [^68^Ga]Ga-ATH001 may permit repeated, longitudinal imaging in individuals, subject to applicable regulatory and ethical requirements.

Furthermore, subjects tolerated the tracer well with no adverse effects related to administration of up to 70 µg precursor peptide (≈ 0.9 µg/kg), indicating a suitable safety profile.

## Conclusion

[^68^Ga]Ga-ATH001 demonstrates a favorable dosimetry and safety profile, supporting further clinical development as a repeatable, non-invasive PET marker of active tissue fibrosis. [^68^Ga]Ga-ATH001 PET enables conceptually novel longitudinal studies on PDGFRβ tissue expression in health and disease.

## Supplementary Information

Below is the link to the electronic supplementary material.

Supplementary file1 (DOCX 179 KB)

## Data Availability

The datasets used and/or analyzed during the current study are available from the sponsor on reasonable request and appropriate ethical approval.
