# Supplementary material for: Biodistribution and radiation dosimetry of the PDGFRβ targeting PET tracer [68Ga]Ga-ATH001
Source: Eur J Nucl Med Mol Imaging. 2026 Sep 8;53(13):8508–13. doi: 10.1007/s00259-026-08167-9 (PMC13727816; doi:10.1007/s00259-026-08167-9)
Supplement: Supplementary file 1 [file 259_2026_8167_MOESM1_ESM.docx]

**Supplementary Data**

**Biodistribution and radiation dosimetry of the PDGFRβ targeting PET tracer [^68^Ga]Ga-ATH001**

Mark Lubberink et al

**Supplementary Figures and Tables**

**Supplementary Table S1:** List of organs delineated for dosimetry assessment and details regarding segmentation methods.

| **No.** | **Organ** | **Delination** | **Delineated on** | **Method** |
| --- | --- | --- | --- | --- |
| **1** | **Liver** | Whole organ delineation | Axial PET / Axial MRI Water coreg. to PET. | Manual. (Corrected for spillover from kidneys) |
| **2** | **Kidneys** | Whole organ delineation | Axial PET | Isocontouring |
| **3** | **Heart wall** | Partial delineation. | Axial PET / Axial MRI Water coreg. to PET. | Manual. (Excluded regions close to liver) |
| **4** | **Lungs** | Whole organ delineation | Axial MRI InPhase, coreg. to PET | Isocontouring. (Excluded regions close to liver and heart) |
| **5** | **Spleen** | Whole organ delineation | Axial PET | Manual. (Corrected for spillover from kidneys) |
| **6** | **Red Marrow** | Partial delineation | Axial PET | Manual delineation of vertebral body (T6-T9) |
| **7** | **Aorta** | Partial delineation | - Dynamic PET: Axial PET on 4-10 Avg frames  -Whole-body scans: Axial SSFSE coreg | Circle VOI 6mm x20 slices (Upper limit: top of T6 vertebrae) |
| **8** | **Salivary glands** | Whole organ delineation | Axial PET | Isocontouring |
| **9** | **Thyroid** | Whole organ delineation | Axial PET | Isocontouring |
| **10** | **Prostate** | Whole organ delineation | Axial PET | Isocontouring |
| **11** | **Cortical bone** | Partial delineation | Axial MRI Water coreg. to PET. Location for Dynamic on arm, for WB on femur | Arm (for dynamic PET):  10 mm outer VOI. 5mm inner VOI substracted. Isocontouring applied to remaining volume  Femur:  15 mm outer VOI. 7mm inner VOI substracted. Isocontouring. 10 slices |
| **12** | **Pancreas** | Partial delineation (Body and tail) | Axial PET / Axial MRI Water coreg. to PET. | Isocontouring |
| **13** | **Gallbladder** | Partial delineation | Axial MRI Water coreg. to PET. | Manual |
| **14** | **Urinary bladder contents** | Full delineation | Axial PET | Isocontouring |
| **15** | **Breasts including skin** | Partial delineation | Axial MRI Water coreg. to PET. | Manual |
| **16** | **Trabecular bone** | Partial delineation (Femur) | Axial MRI Water coreg. to PET. | Manual |
| **17** | **Muscle** | VOI Sphere | Axial MRI Water coreg. to PET. | VOI Sphere (25mm) – gluteus. (biceps on dynamic) |
| **18** | **Stomach content** | VOI Sphere | Axial MRI Water coreg. to PET. | VOI Sphere (15mm) |
| **19** | **Brain** | VOI Sphere | Axial MRI Water coreg. to PET. | VOI Sphere (25mm) |
| **20** | **Testes** | VOI Sphere | Axial MRI Water coreg. to PET. | VOI Sphere (10mm) |
| **21** | **Ovaries** | Partial delineation | Axial MRI Water coreg. to PET. | Manual |
| **22** | **Uterus** | Full delineation | Axial PET | Isocontouring |
| **23** | **Small intestines content** | VOI Sphere | Axial MRI Water coreg. to PET. | VOI Sphere (6mm) |
| **24** | **Upper large intestine content** | VOI Sphere | Axial MRI Water coreg. to PET. | VOI Sphere (6mm) |
| **25** | **Lower large intestine content** | VOI Sphere | Axial MRI Water coreg. to PET. | VOI Sphere (6mm) |

**Supplementary Table S2:** Effective half-lives and extrapolated fractions for [^68^Ga]Ga-ATH001.

| Organ | Effective half-life  (mean ± SD, min) | Extrapolated fraction  (mean ± SD, %) |
| --- | --- | --- |
| Brain | 115 ± 165 | 19 ± 28 |
| Breast | 90 ± 14 | 22 ± 5 |
| Cortical bone | 65 ± 50 | 14 ± 17 |
| Gallbladder | 56 ± 12 | 9 ± 4 |
| Heart Wall | 60 ± 23 | 11 ± 9 |
| Kidneys | 91 ± 5 | 23 ± 3 |
| Liver | 39 ± 4 | 3 ± 1 |
| LLI | 51 ± 23 | 9 ± 8 |
| Lungs | 34 ± 15 | 3 ± 3 |
| Ovaries | 66 ± 33 | 14 ± 13 |
| Pancreas | 36 ± 5 | 3 ± 1 |
| Prostate | 60 ± 3 | 13 ± 2 |
| Red Marrow | 44 ± 13 | 6 ± 5 |
| Salivary Glands | 50 ± 4 | 9 ± 2 |
| SI | 44 ± 7 | 5 ± 2 |
| Spleen | 40 ± 7 | 4 ± 3 |
| Stomach contents | 46 ± 30 | 7 ± 9 |
| Testes | 86 ± 1 | 24 ± 1 |
| Thyroid | 44 ± 2 | 6 ± 1 |
| Trabecular Bone | 62 ± 6 | 13 ± 2 |
| ULI | 55 ± 11 | 9 ± 4 |
| Urinary Bladder contents | 127 ± 77 | 33 ± 20 |
| Uterus | 73 ± 4 | 17 ± 1 |

**Supplementary Table S3:** Time-integrated activity coefficients for [^68^Ga]Ga-ATH001.

| Organ | Female (N=2)  (mean ± SD, h) | Male (N=4)  (mean ± SD, h) |
| --- | --- | --- |
| Brain | 0.002 ± 0.000 | 0.002 ± 0.000 |
| Breast | 0.004 ± 0.000 |  |
| Cortical bone | 0.022 ± 0.005 | 0.031 ± 0.004 |
| Gallbladder | 0.004 ± 0.001 | 0.003 ± 0.001 |
| Heart Wall | 0.017 ± 0.003 | 0.020 ± 0.005 |
| Kidneys | 0.372 ± 0.017 | 0.322 ± 0.032 |
| Liver | 0.166 ± 0.003 | 0.207 ± 0.054 |
| LLI | 0.003 ± 0.001 | 0.003 ± 0.002 |
| Lungs | 0.016 ± 0.002 | 0.018 ± 0.006 |
| Ovaries | 0.000 ± 0.000 |  |
| Pancreas | 0.023 ± 0.000 | 0.024 ± 0.004 |
| Prostate |  | 0.002 ± 0.000 |
| Red Marrow | 0.105 ± 0.010 | 0.105 ± 0.003 |
| Salivary Glands | 0.011 ± 0.001 | 0.010 ± 0.001 |
| SI | 0.041 ± 0.001 | 0.041 ± 0.002 |
| Spleen | 0.065 ± 0.007 | 0.058 ± 0.008 |
| Stomach contents | 0.003 ± 0.001 | 0.008 ± 0.004 |
| Testes |  | 0.001 ± 0.000 |
| Thyroid | 0.003 ± 0.000 | 0.004 ± 0.000 |
| Trabecular Bone | 0.014 ± 0.003 | 0.018 ± 0.006 |
| ULI | 0.010 ± 0.003 | 0.010 ± 0.008 |
| Urinary Bladder contents | 0.040 ± 0.015 | 0.039 ± 0.011 |
| Uterus | 0.011 ± 0.001 |  |
| Remainder of the body | 0.699 ± 0.027 | 0.705 ± 0.105 |
